# Large language models in bioinformatics: a comprehensive survey

**DOI:** 10.3389/fgene.2026.1797863

**Published:** 2026-08-24

**Authors:** Zhigang Meng, Zhikai Yang, Mingxun Zhu, Jianzhen Xu

**Affiliations:** 1 School of Computer Science and Engineering, Changsha University, Changsha, China; 2 Department of Computer Science, College of Computer, Hunan University of Technology, Zhuzhou, China; 3 Department of Big Data Service and Management, School of Economics and Management, Changsha Normal University, Changsha, China; 4 Shantou University Medical College, Shantou, China

**Keywords:** bioinformatics, drug discovery, genomics, large language models, multimodal learning, protein structure prediction

## Abstract

The emergence of foundation models with trillion-level parameters has redefined the landscape of artificial intelligence. Various fields are developing their own large-scale models, which can solve many problems within the field and improve work efficiency. Biological large-scale models are a cross-disciplinary research field that combines mathematics, computer science, and biology, aiming to simulate and understand the structure, function, and dynamic changes of biological systems through the establishment of complex computational models. This field covers multiple levels such as biological pathways, population dynamics, protein folding, etc., providing us with tools for deep exploration of the mysteries of life and applications in medicine, ecology, and other fields. This article reviews the background and research status of biological large-scale models, and discusses future directions. Large language models (LLMs) and other large-scale foundation models have rapidly advanced in recent years, enabling powerful representation learning and generation across text, sequences, and multimodal data. In bioinformatics and biomedicine, these models are increasingly used to analyze genomic sequences, infer protein properties and structures, support drug discovery, and integrate heterogeneous biomedical evidence. This survey reviews the basic principles of LLMs and summarizes representative applications in (i) gene and genome sequence analysis, (ii) protein structure and function prediction, and (iii) drug design, including virtual screening and personalized medicine. We also discuss emerging multi-model modeling approaches, as well as key challenges such as data quality and privacy, interpretability, generalization to new organisms and tasks, and responsible deployment in health-related settings. Finally, we outline future directions for developing reliable, scalable, and explainable bioinformatics foundation models.

## 1 Introduction

With the rise of deep learning, large language models (LLMs) have transformed natural language processing by learning general-purpose representations from massive corpora. These models, which can process and generate large volumes of text data, have the potential to enhance various areas of bioinformatics. In this article, we provide a comprehensive overview of the principles of LLMs and their recent applications in genome sequence analysis, protein structure prediction, drug discovery, and other areas of bioinformatics research.

The development of language models has undergone several transformative stages over the past decade. The early era (2013–2017) was characterized by distributed representation methods such as Word2Vec (Mikolov et al., 2013), GloVe (Pennington et al., 2014), and Doc2Vec (Le and Mikolov, 2014), which enabled machines to capture semantic relationships between words through dense vector embeddings. These methods, while groundbreaking, were limited by their inability to capture contextual information and long-range dependencies in text.

The pre-trained language model era (2018–2019) marked a significant paradigm shift with the introduction of BERT (Devlin et al., 2018) and the GPT series (Radford et al., 2018; Radford et al., 2019). BERT’s bidirectional transformer architecture achieved unprecedented performance on various natural language understanding tasks, while GPT demonstrated the potential of autoregressive language modeling at scale. These models established the foundation for transfer learning in NLP, showing that pre-training on large corpora followed by task-specific fine-tuning could substantially improve performance across diverse applications.

The large language model era (2020-present) has been defined by unprecedented scale and capability. GPT-3 (Brown et al., 2020) with 175 billion parameters demonstrated emergent abilities and few-shot learning capabilities. Subsequent models including PaLM (Chowdhery et al., 2023), LLaMA (Touvron et al., 2023), and GPT-4 (OpenAIet al., 2023) have further pushed the boundaries of what language models can achieve. Notably, AlphaFold2 (Jumper et al., 2021) demonstrated that transformer-based architectures could revolutionize protein structure prediction, bridging the gap between language models and biological sequence analysis. More recently, models such as ESM-2 (Lin et al., 2023) and ProteinMPNN (Dauparas et al., 2022) have specifically targeted protein sequence-structure relationships, while DNABERT-2 (Zhou et al., 2023) and Nucleotide Transformer (Dalla-Torre et al., 2024) have advanced genomic sequence understanding.

**FIGURE 1:**
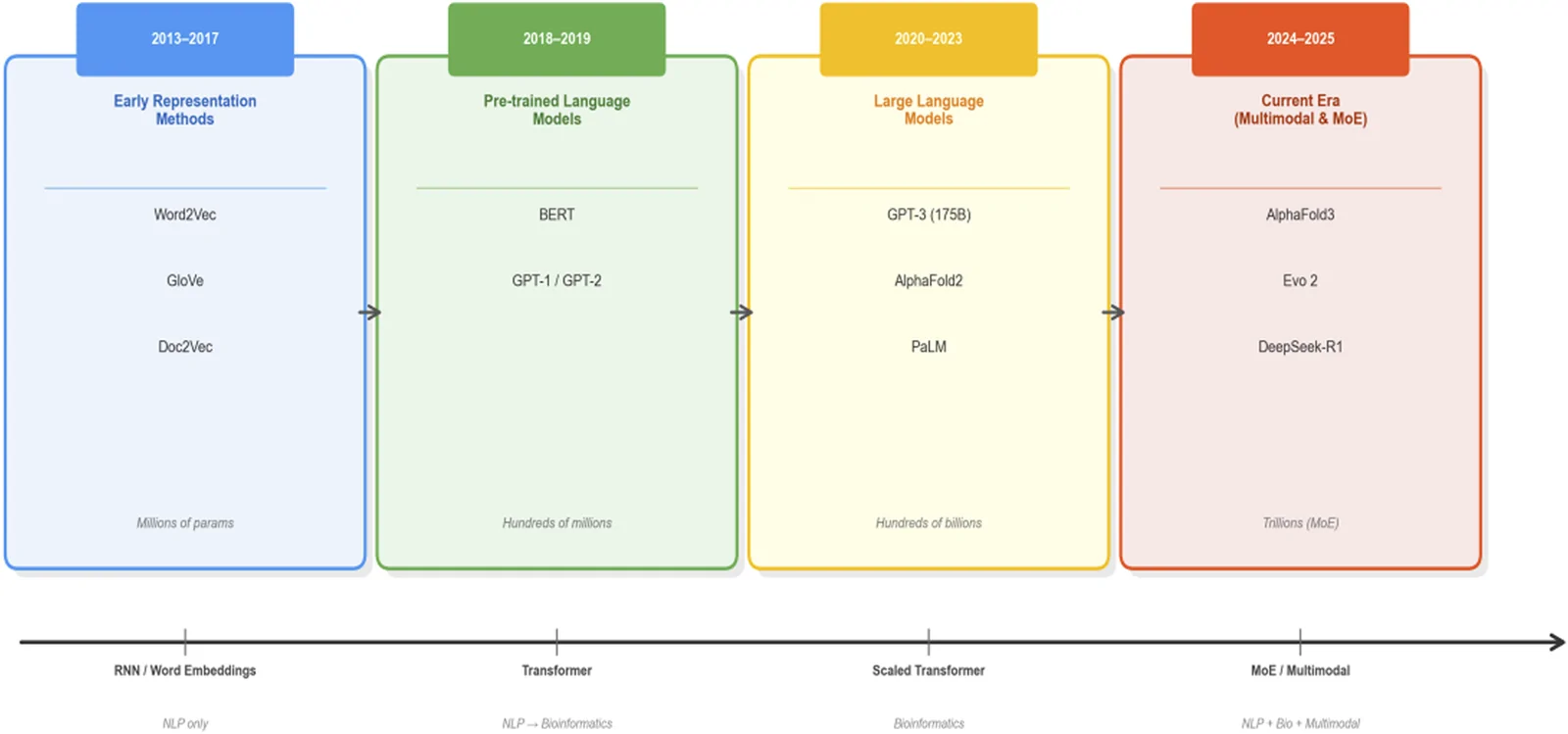
Evolution of Large Language Models (2013–2025). The timeline illustrates four distinct eras as described in the text, showing the progression of parameter scale, architectural innovations, and application domains from NLP to Bioinformatics and Multimodal integration.

Figure 1 illustrates the timeline of four distinct eras: ① Early Representation Methods Era (2013–2017) featuring Word2Vec, GloVe, and Doc2Vec with million-scale parameters; ② Pre-trained Language Models Era (2018–2019) with BERT and GPT-1/2 introducing transformer architectures; ③ Large Language Models Era (2020–2023) characterized by billion-scale parameters and bioinformatics applications; ④ Current Era (2024–2025) featuring trillion-scale sparse models with multimodal capabilities. The progression shows increasing parameter scale (millions → billions → trillions), architectural innovations (RNN → Transformer → MoE), and application expansion (NLP → Bioinformatics → Multimodal).

Among notable breakthroughs in this era, DeepSeek-R1 (Guo et al., 2025) demonstrated remarkable reasoning capabilities approaching frontier model performance with open-source weights, reported in Nature. AlphaFold3 (Abramson et al., 2024), published in Nature in 2024, extended structure prediction beyond proteins to DNA, RNA, and small molecules, further expanding the scope of LLM-based bioinformatics applications. Meanwhile, Evo (Nguyen et al., 2024), a genomic foundation model trained on prokaryotic genomes at single-nucleotide resolution, was published in Science, enabling zero-shot prediction of fitness effects and generation of novel functional sequences across diverse genomic elements. Building upon this foundation, Evo 2 (Brixi et al., 2026), released in 2025 and trained on 9.3 trillion DNA base pairs spanning all domains of life, extended these capabilities to eukaryotic and viral genomes, demonstrating state-of-the-art performance in variant effect prediction and *de novo* genome design at unprecedented scale.

Recent LLMs generally have the following characteristics:

1. Model scale: The number of parameters owned by the training model (Brown et al., 2020; Ouyang et al., 2022).
2. Model complexity: The number of layers and neurons in the model.
3. The scale of training data: The model needs to learn the features of the data through a large amount of training data.
4. Self-Supervised Learning: Can be trained on unmarked text (Gulshan et al., 2016; Zhang et al., 2019).

In recent years, with the accumulation of biological data and the development of LLMs, the problem of studying biological LLMs lies in how to obtain large-scale labeled data (Yuan et al., 2021). The development of reinforcement learning (Christiano et al., 2017) and self-supervised learning has significantly reduced the reliance on large-scale manual annotation. However, expert-curated labels remain indispensable for constructing high-quality benchmarks, validating model predictions, and ensuring clinical reliability. Self-supervised paradigms should therefore be viewed as complementary to, rather than a complete replacement for, rigorous data curation pipelines. LLMs can be applied to protein ligand docking and virtual screening, predicting protein ligand interactions, and identifying potential drug targets. It is also possible to more accurately predict the three-dimensional structure of proteins by integrating multi-level data. Because of the advantages of LLMs in processing large-scale data and learning complex patterns, LLMs can not only analyze genomes and gene expression profiles, but also perform non coding RNA analysis, and so on. The application in gene sequence analysis is becoming increasingly common.

Large multimodal models (LMMs) are an artificial intelligence technology with broad application prospects, which can achieve the fusion processing of multiple modal information (such as images, speech, text, etc.), thereby improving the intelligence level of artificial intelligence systems. LMMs can process not only single modal information, but also multi-modal information. Common multimodal pre training tasks include video text matching (Radford et al., 2021; Jia, 2021), audio text matching, cross modal generation (Lin et al., 2023; Liang et al., 2020), image video association, etc. The working principle of LMMs is to fuse information from different modalities through deep neural network models. During the model training process, LMMs will learn the correlation between different modalities, thereby achieving effective fusion of multimodal information. This fusion method can not only improve the intelligence level of the model, but also enhance its generalization ability (Yosinski et al., 2014), making the model more accurate and efficient in handling practical problems.

As biological data continues to grow, a major bottleneck is not only the availability of labeled datasets but also the integration of heterogeneous sources (e.g., sequences, structures, images, and clinical text). Advances in self-supervised learning and reinforcement learning have reduced reliance on manual annotation and enabled models to learn from large-scale unlabeled biomedical data. These capabilities support applications such as protein–ligand docking and virtual screening, prediction of protein–ligand interactions, and improved inference of protein three-dimensional structure by integrating multiple levels of evidence. LLMs are also increasingly applied to genome and transcriptome analysis, including promoter prediction, splicing-site detection, non-coding RNA analysis, and metagenomic classification (Avsec, 2021; Zeng and Li, 2022).

## 2 Gene sequence analysis

Gene sequence analysis is a fundamental branch of bioinformatics that focuses on the study of DNA, RNA, and protein sequences. The emergence of high-throughput sequencing technology has driven the development of gene sequences, allowing us to extract various data to gain a deeper understanding of the basic characteristics and mechanisms of life. Through gene sequence analysis, it is possible to investigate cellular functions, protein structures, and the genomic locations of genes in living organisms. In agriculture, gene sequence analysis plays an important role in crop improvement, yield enhancement, and environment adaptation. From an ecological perspective, it facilitates the study of genetic diversity, evolutionary relationships among species, and the inference of gene evolutionary histories. These applications collectively highlight the central role of gene sequence analysis in the ongoing development of bioinformatics (Avsec, 2021; Zeng and Li, 2022).

### 2.1 Application of deep learning models in gene sequence classification

Deep learning models are an advanced form of artificial neural networks (ANNs) that have achieved significant success in various tasks in recent years, including image recognition, speech recognition, natural language processing, etc. They are also widely used in gene sequence analysis (Zhang et al., 2019; Ching, 2018).

DeepMicrobes (Liang et al., 2020) is a deep learning-based approach designed for microbial classification. Compared to advanced tools at the time in species and genus identification, it has similar accuracy in large-scale estimation. DeepMicrobes helps to effectively study the unknown roles of microbial species, providing new methods for the classification of metagenomics. But there are also issues such as relying on complex models, slow runtime, and high memory requirements. MetaTransformer (Wichmann et al., 2023) is a deep learning metagenomic analysis tool based on self attention mechanism, which can achieve effective parallelization. While solving the above problems, it outperforms DeepMicrobes in terms of species and genus classification ability, training speed, and inference speed. The results have demonstrated the feasibility and effectiveness of applying the Transformer (Vaswani et al., 2017) model to metagenomic classification.

ConF (Teragawa and Wang, 2023) is a deep learning model for non-coding RNA (ncRNA) family classification that surpasses existing algorithms in terms of accuracy, recall, precision, and F1 score. By integrating BiLSTM (Sun et al., 2015), CNN (LeCun et al., 1998), and multi-head cross-attention mechanisms, ConF effectively captures both sequence and structural features of RNA.

In gene promoter prediction, traditional approaches typically use non-promoter genomic regions as negative samples. DeePromoter (Oubounyt et al., 2019) introduces a novel strategy by constructing more challenging negative samples derived from promoter sequences. This approach enhances the model’s discriminative capability and significantly reduces false positives, achieving superior performance compared with prior methods. Nevertheless, issues such as limited cross-species generalization, high model complexity, strong dependence on negative samples, and limited biological interpretability remain. To overcome these limitations, iPro-WAEL (Zhang et al., 2022), a weighted average ensemble learning model, was proposed for multi-species promoter identification.

#### 2.1.1 Model architecture comparison and selection rationale

Before delving into specific applications, it is essential to understand the architectural distinctions among different deep learning models and their respective utilities in bioinformatics tasks (Zhang et al., 2019).

- CNNs rely on local receptive fields and weight sharing, making them highly effective for short-range motif scanning (e.g., transcription factor binding sites) but less suited for distal dependencies.
- RNNs process tokens sequentially with hidden state propagation, which inherently models temporal order but suffers from vanishing gradients and limited parallelization. They are still utilized when computational resources are constrained.
- GNNs operate on explicit graph topologies via message passing, excelling in relational networks like protein-protein interactions and regulatory network analysis.
- LLMs/Transformers employ global pairwise self-attention with positional encoding, enabling context-aware token representations without recurrence. They are best suited for cross-species transfer, long-range genomic interactions, and zero-shot prediction tasks (Ching, 2018).

### 2.2 The application of transformer model in gene sequence prediction

**FIGURE 2:**
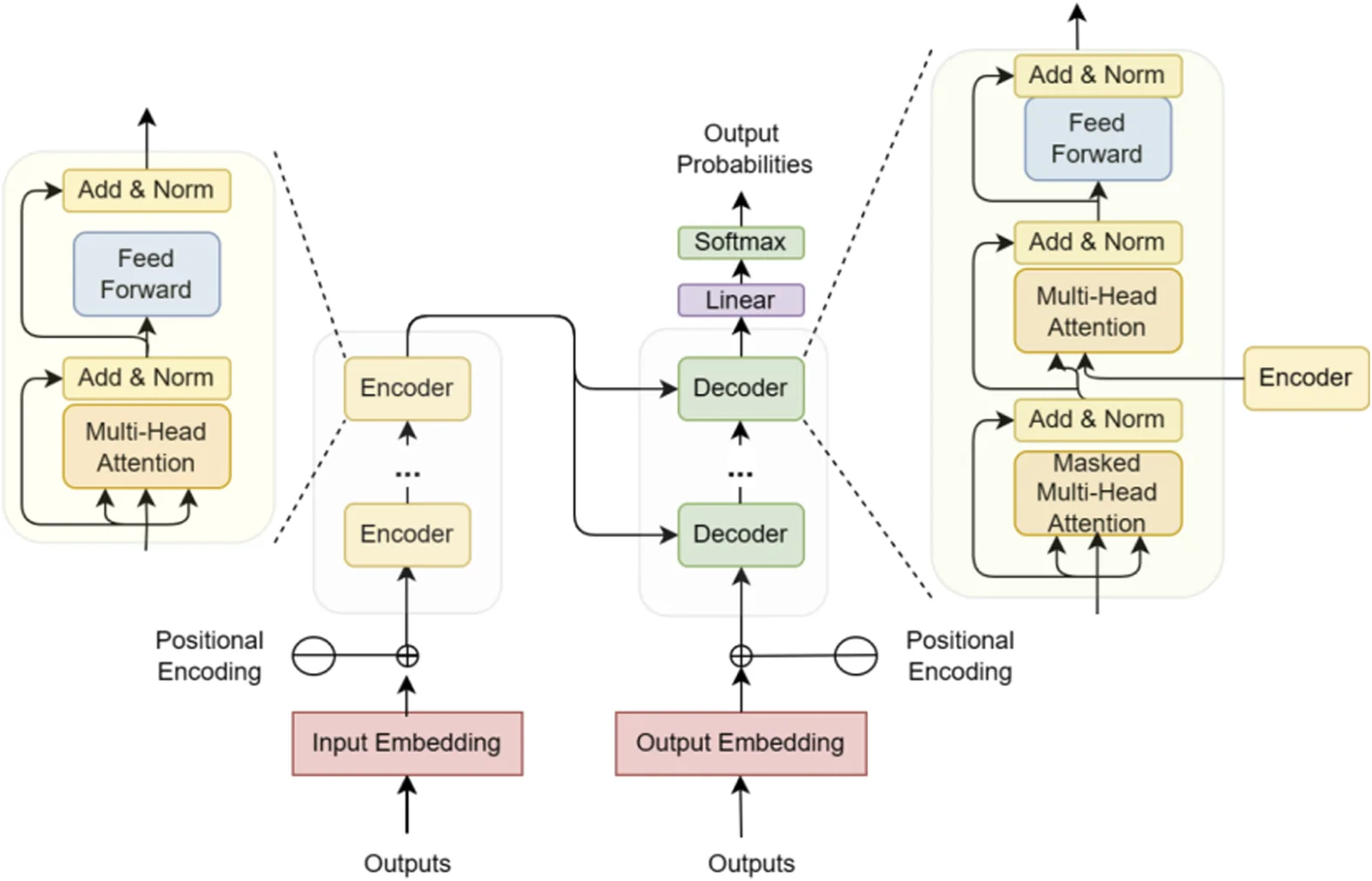
Schematic architecture of the Transformer model. Key components include multi-head self-attention mechanisms, positional encodings, and feed-forward neural networks. This parallelizable architecture forms the computational backbone of modern bioinformatics LLMs (Vaswani et al., 2017).

The Transformer model is a model that utilizes attention mechanisms to improve model training speed. Its performance and accuracy are higher than previous RNN models, so it is widely used in various fields (as illustrated in Figure 2). Prior to the Transformer, sequence modeling in genomics relied heavily on RNNs and LSTMs (Rumelhart et al., 1986; Hochreiter and Schmidhuber, 1997), which process nucleotides sequentially. This architecture suffers from vanishing gradients over long genomic contexts, limited parallelization during training, and a constrained effective receptive field. In contrast, the Transformer architecture leverages self-attention to compute pairwise interactions across the entire sequence in parallel, enabling direct modeling of distal regulatory elements (e.g., enhancer-promoter loops). Additionally, Transformers utilize learned positional embeddings and tokenization strategies (e.g., k-mer or byte-pair encoding) that facilitate transfer learning across diverse genomic tasks without task-specific architectural redesign.

Transformer-based models have demonstrated exceptional capacity in handling unlabeled genomic sequences. The Transformer based genome prediction model GPformer (Wu et al., 2024) was first proposed in genome prediction (GP) to solve the problems of limited information flow and imbalanced data in traditional GP methods. A knowledge guided module (KGM) was also introduced to improve prediction performance. BERT (Devlin et al., 2018) is a transformer-based representation model. After its widespread application, DNABERT (Ji et al., 2021) was trained on the BERT model for gene sequence analysis, achieving state-of-the-art performance in predicting promoters, splicing sites, and transcription factor binding sites. It can also directly visualize the importance and semantic relationships of nucleotide levels in input sequences. In another study, DNABERT was fine tuned to identify long non coding RNAs (lncRNAs) (Danilevicz et al., 2023) from plant genome sequences. BERT-5 mC (Wang S. et al., 2023; Wang et al., 2021) is an interpretable model based on BERT that can predict the 5-methylcytosine (5 mC) site on DNA, achieving an AUROC of 0.966. Building upon DNABERT (Ji et al., 2021), DNABERT-2 (Zhou et al., 2023) addresses the limitations of k-mer tokenization by adopting a multi-species byte-pair encoding (BPE) tokenization strategy. Similarly, the Nucleotide Transformer (Dalla-Torre et al., 2024) demonstrated that pre-training foundation models on large collections of genomic sequences from diverse species enables remarkable zero-shot performance.

### 2.3 Research on training models using large-scale genomic data

The current models have evolved from billions of parameters to trillions of parameters (for example, Switch Transformer already contains 160 billion parameters) (Fedus et al., 2022), and even trillions of data tokens (for example, MPT-30B-Instrument pre trained 1 trillion data tokens) (Hoffmann et al., 2022). Although there is no clear statement that the larger the model’s size and data, the better, GPT1 to GPT3.5 (Radford et al., 2018; Radford et al., 2019; Brown et al., 2020; Ouyang et al., 2022) have made significant improvements in terms of task versatility and multimodal processing with the increase of parameters and training data (Fedus et al., 2022; Hoffmann et al., 2022).

When studying how to handle large datasets, PROTRAIT (Wang Z. et al., 2023) learns the complex relationship between DNA sequences and chromatin accessibility through ProdDep Transformer Encoder. When studying how to improve the accuracy and efficiency of virus classification, a BERT model specifically designed for DNA analysis was proposed (Ren et al., 2020). The recent CellPolaris (Feng et al., 2026) model was trained based on the Graph based Recurrent Neural Network (GRN) model, using the RNA seq dataset for training. GeneCompass (Yang et al., 2024) is a pretrained model trained on a single cell transcriptome dataset comprising 120 million human and rodent cells, enabling cross-species analysis of gene regulatory mechanisms.

### 2.4 A gene sequence analysis method combining multimodal data

Multimodal data consists of multiple forms of information and are increasingly used in deep learning and artificial intelligence. Integrating multimodal data enables more comprehensive feature representations, improves model robustness and generalization, and promotes cross-disciplinary research (Zhang et al., 2019; Ching, 2018). RNA degformer (He et al., 2023) is a novel deep learning model that combines prediction results generated by multiple models as additional features during training. Table 1 summarizes representative applications of large language models (LLMs) in bioinformatics.

**TABLE 1:** The application of LLMs in the field of bioinformatics.

Category	Model	Architecture/Base	Access	Primary task/Evaluation
Genomics	DNABERT (Ji et al., 2021)	BERT	Open	Promoters and splice-site prediction
	BERT-5mC (Wang S. et al., 2023)	BERT	Open	DNA 5 mC site identification
	GeneCompass (Yang et al., 2024)	Foundation	Open	Cross-species gene regulation
Proteomics	AlphaFold2 (Jumper et al., 2021)	Transformer	Open	End-to-end 3D structure prediction
	ProteinBERT (Brandes et al., 2022)	BERT	Open	Global protein representation learning
	SecProCT (Du et al., 2021)	Transformer	Closed	Human secreted protein identification
Drug Design	TransDTI (Kalakoti et al., 2022)	Transformer	Closed	DTI and drug recommendation
	DrugAssist (Ye et al., 2023)	LLM-based	Open	Interactive molecule optimization
	DNABERT-2 (Zhou et al., 2023)	BERT + BPE	Open	Multi-species genomic sequence understanding
	Nuc. Transformer (Dalla-Torre et al., 2024)	Transformer	Open	Regulatory annotation
Proteomics	ESM-2 (Lin et al., 2023)	Transformer	Open	Protein function prediction
	ProteinMPNN (Dauparas et al., 2022)	MPNN	Open	Protein sequence design
	ProtTrans (Elnaggar et al., 2021)	BERT/GPT/T5	Open	Secondary structure prediction
Drug Design	TxGNN (Huang et al., 2024)	GNN	Open	Zero-shot drug prediction
	BioMedGPT (Luo et al., 2023)	Multimodal GPT	Open	Drug-target interaction

Table 1 reveals several emerging trends in the application of LLMs to bioinformatics. First, there has been a clear progression from task-specific deep learning models toward general-purpose foundation models capable of zero-shot or few-shot transfer. Second, the genomics domain has witnessed explosive growth following DNABERT (Ji et al., 2021), with DNABERT-2 (Zhou et al., 2023) and the Nucleotide Transformer (Dalla-Torre et al., 2024) demonstrating that scaling and improved tokenization strategies yield substantial performance gains across diverse genomic tasks. Third, in the proteomics domain, models such as ESM-2 (Lin et al., 2023) and ProteinMPNN (Dauparas et al., 2022) have established that language model-derived protein representations can match or exceed physics-based methods. Fourth, drug design is increasingly leveraging conversational and multimodal LLM frameworks.

Specific disease areas have benefited particularly from these multimodal approaches. In the field of Parkinson’s disease, a new brain disease multimodal data analysis model CERNNE (Bi et al., 2021) was proposed based on fMRI and SNP data. A multimodal integration framework called Pathomic Fusion (Chen, 2020) was designed in the field of cancer. Later, a new hierarchical multimodal fusion method HFBSurv (Li et al., 2022) was proposed.

## 3 Protein structure prediction

Protein structure prediction focuses on the three-dimensional conformations of proteins and is fundamental to understanding disease mechanisms and biological processes. Traditional experimental approaches of protein structure determination are often time-consuming, labor-intensive, and costly. The rapid advancement and widespread adoption of LLMs have demonstrated significant potential to address these challenges.

### 3.1 Applicability of LLMs to protein structure prediction

A critical question is whether LLMs are suitable for protein structure prediction (Kuhlman and Bradley, 2019). For end-to-end 3D coordinate prediction, pure language models face fundamental limitations because they operate on discrete token sequences rather than continuous 3D space. This is why AlphaFold2 (Jumper et al., 2021) employs a sophisticated architecture combining attention-based sequence processing with explicit structural modules. However, LLMs excel in related tasks that benefit from evolutionary sequence analysis (Petti et al., 2023). Protein language models such as ESM-2 (Lin et al., 2023) learn rich representations from multiple sequence alignments (MSAs). Recent hybrid approaches demonstrate that the most effective architectures integrate transformer-based sequence encoding with graph neural networks or equivariant networks (Jing et al., 2021; Xing et al., 2022).

### 3.2 Application of deep learning in protein structure prediction

The DEDAL (Llinares-López et al., 2023) model has emerged in protein sequence alignment. An efficient method assisted by the ESM (Zhang Y., 2023) model was introduced to predict thermal stability changes (Cao et al., 2019). SecProCT (Du et al., 2021) integrates capsule networks and Transformers. Research on LLMs, such as BepiPred-3.0 (Clifford et al., 2022), trains on large datasets of protein sequences and structures. Beyond direct application, ESM-2 (Lin et al., 2023) embeddings have been widely adopted as sequence representations. ProtTrans (Elnaggar et al., 2021) demonstrated that protein language model embeddings can effectively replace handcrafted features. MolBERT (Fabian et al., 2020) applied BERT-style pre-training to molecular SMILES strings.

### 3.3 Principles and impact of transformative models: the AlphaFold breakthrough

**FIGURE 3:**
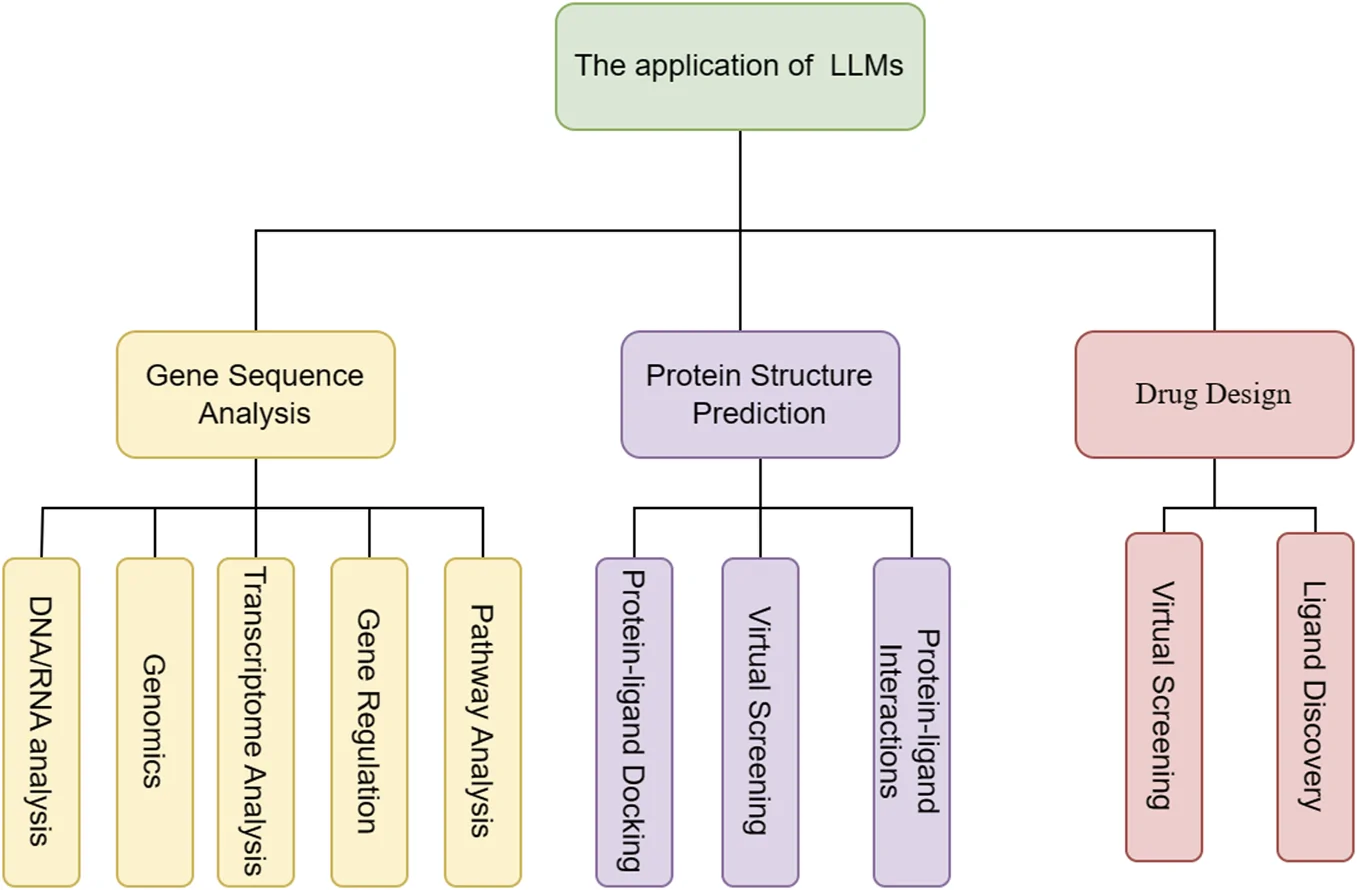
The applications of LLMs in the field of bioinformatics. The figure organizes representative methodologies into four thematic blocks (1): Drug Design (2), Protein Structure Prediction (3), Gene Sequence Analysis, and (4) Multimodal Integration.

As illustrated in Figure 3, LLMs have been applied across multiple core areas of bioinformatics, encompassing drug design, protein structure prediction, genome analysis, and clinical applications. AlphaFold (Jumper et al., 2021) demonstrated groundbreaking accuracy in protein structure prediction, achieving top performance in the 13th and 14th CASP competitions. The methodologies in Figure 3 are organized according to two criteria: (1) biological application domain; and (2) methodological maturity and impact level, categorized based on four quantitative/qualitative criteria: (i) benchmark performance (e.g., CASP/CAMEO scores), (ii) community adoption (citation count, open-source availability), (iii) integration into standard pipelines, and (iv) preclinical/clinical translation status.

On CASP14, AlphaFold 2 achieved a significant breakthrough, reaching 92.4/100. AlphaFold2 mainly consists of two parts: neural network Evoformer and structural module. In Evoformer, the main approach is to combine graph networks and multiple sequence alignment (MSA).

### 3.4 Protein structure prediction combining deep learning and traditional biophysical methods

The Potts model, a statistical energy-based framework derived from statistical physics, captures coevolutionary constraints (Guerois et al., 2002; Alford et al., 2017; Kamisetty et al., 2013). The MSA Transformer (Sgarbossa et al., 2023; Cai et al., 2021) model showed that sequences generated by large product families had similar or better characteristics than the Potts model. ResPRE (Li et al., 2019), a method that combines precise matrices and deep residual neural networks, has a higher accuracy in top-L remote contact prediction than coevolution-based methods (Jones and Kandathil, 2018; Seemayer et al., 2014; Wang et al., 2017). Bingo (Ma et al., 2024) combined LLM and GNN with adversarial training.

### 3.5 Research on end-to-end protein structure prediction model

End to end protein structure prediction directly predicts three-dimensional structures from protein sequences. DeepECA (Fukuda and Tomii, 2020) has been proposed to address the potential decrease in statement results caused by rich sequences. RGN2 (Chowdhury et al., 2022), a new deep learning method for predicting protein structure based on a single protein sequence. It demonstrates superior performance to AlphaFold2 in single-sequence monomer prediction tasks on benchmarks such as CAMEO (Chowdhury et al., 2022), where AlphaFold2’s performance degrades without multiple sequence alignment (MSA) inputs. Another model is SecProCT (Du et al., 2021), and a new algorithm trRosettaX Single (Wang et al., 2022) has emerged.

## 4 Drug design

Drug design refers to the process of discovering, designing and optimizing novel therapeutic compounds (Bai et al., 2015; Wüthrich, 2001; Xu et al., 2021). Today, drug design plays a critical role in modern medicine.

### 4.1 Architectural considerations in drug design

For molecular property prediction and virtual screening, the choice between CNNs, GNNs, and LLMs depends on the representation of molecular data. GNNs naturally handle molecular graphs (Gomez-Bombarelli et al., 2018). LLMs have shown remarkable capabilities in learning from SMILES strings. For drug-target interaction (DTI) prediction (Stärk et al., 2022), recent LLM-based approaches leverage pre-trained protein language models and chemical language models to encode both targets and ligands into a shared embedding space.

### 4.2 Applications of large language models in virtual screening and ligand discovery

As shown in the Drug Design block of Figure 3, Virtual screening mainly uses computer simulation analysis to pre-screen a large number of compound libraries before biological activity screening, with the aim of quickly identifying small molecules that are most likely to bind to specific drug targets.

In contrast, although virtual screening methods are also very useful in drug design, they may not be as good as large language models (LLMs) in terms of data processing capabilities, innovation, and multi-tasking. Virtual screening usually relies on known biological target structures and existing compound libraries, while large language models (LLMs) can predict and generate new compound structures by learning a large amount of biomedical data, and can even perform drug design without a clear target structure.

In terms of virtual screening (Zhang J., 2023), the language model has powerful natural language processing capabilities. Through training, the language model can learn the interaction pattern between the compound and the target. In terms of ligand discovery, LLMs can analyze the interaction between the ligand and the target. In addition, by using large language models (LLMs) to analyze the molecular characteristics and mechanism of action of existing drugs, its potential therapeutic effect on other diseases can be discovered, thus achieving drug repurposing (i.e., identifying new therapeutic uses for existing approved drugs) (Corso et al., 2023; Shao et al., 2022).

DataDTA (Zhu et al., 2023) is a novel deep learning-based predictor. The ConPlex (Singh et al., 2023) model combines pre-trained protein language models (PLMs) and contrastive learning. DrugAssist (Ye et al., 2023) is an interactive molecular optimization model that uses the interactivity and generalization of large language models to optimize molecules through a human-in-the-loop conversational interface (i.e., interactive optimization guided by conversational user feedback). Recent SOTA methods include TxGNN (Huang et al., 2024), ChatDrug (Liu et al., 2023), and BioMedGPT (Luo et al., 2023).

### 4.3 Applications of large language models in personalized drug design based on genomic and transcriptomic data

The core of personalized drug design lies in understanding and utilizing individual genetic variations (Yu et al., 2023) and molecular expression patterns (Jha, 2023). By analyzing genomic data, Large Language Models (LLMs) can help identify gene variants associated with specific diseases. Using genomic and transcriptomic data, LLMs can predict possible drug targets (Kalakoti et al., 2022). LLMs can simulate the interaction between drugs and targets and predict the efficacy and selectivity of drugs (Liu et al., 2021).

The advent of the era of personalized medicine requires us to be able to predict drug response and toxicity based on individual genetic information (Yu et al., 2020). Traditional methods for predicting drug response and toxicity often rely on limited biomarkers and empirical rules, while Large Language Models (LLMs) provide a new perspective, integrating genomic and transcriptomic data through deep learning and natural language processing technology to predict drug response and toxicity more comprehensively and accurately (Liu et al., 2021; Xiong et al., 2021; Jiménez-Luna et al., 2020). Large Language Models are mainly implemented through the following steps: 1. Data integration and feature extraction; 2. Model training and fine-tuning; 3. Drug property encoding; 4. Predictive modeling; 5. Model evaluation and verification. These steps are typically validated against standardized ADMET benchmarks and toxicity datasets (e.g., Tox21) to ensure pharmacological relevance (Liu et al., 2021; Xiong et al., 2021).

## 5 Future outlook

Although the advancement of self-supervised learning technology has reduced the reliance on large-scale data annotation, high-quality, high-dimensional and diverse biological datasets are still scarce. Biomedical data has multimodal characteristics (LeCun, 2022). How to design a model that can effectively integrate these cross-modal information is a difficult problem to be solved.

To build large-scale, high-quality biological datasets, we can leverage the power of reinforcement learning models. By using cutting-edge technologies such as data lakes (centralized repositories that preserve raw heterogeneous biomedical data in native formats, enabling scalable cross-modal querying and large-scale integration) (Hai et al., 2023) and federated learning, we can aggregate data from all parties and train models while fully protecting privacy (Rieke et al., 2020). This will provide us with a rich and secure data foundation to support deeper research. To more effectively process multimodal data, we can develop multimodal learning techniques and integrate advanced algorithms such as Transformer, graph neural networks, and attention mechanisms (Zhang et al., 2019; Ching, 2018). At the same time, ethical considerations must be embedded into the design and deployment of large bioinformatics models from the outset. We firmly believe that only models that comply with regulations and win the public’s trust can promote the real implementation of health applications.

## 6 Conclusion

Biological data are generated from increasingly diverse sources and exist in a wide range of formats (Zhang et al., 2019). As powerful tools for modern biomedical research, large language models have already demonstrated their ability to facilitate deeper biological insight and discovery (Liu et al., 2021; Jiménez-Luna et al., 2020). Looking ahead, LLMs are expected to work in increasingly close partnership with human researchers. With continued innovation and responsible governance, LLMs are poised to make significant contributions to biomedical research, human health, and scientific advancement (Rieke et al., 2020).
